# Collagen and calcium-binding EGF domains 1 is frequently inactivated in ovarian cancer by aberrant promoter hypermethylation and modulates cell migration and survival

**DOI:** 10.1038/sj.bjc.6605429

**Published:** 2009-11-24

**Authors:** C A Barton, B S Gloss, W Qu, A L Statham, N F Hacker, R L Sutherland, S J Clark, P M O'Brien

**Affiliations:** 1 Cancer Research Program, Garvan Institute of Medical Research, Darlinghurst, Sydney, NSW 2010, Australia; 2 Gynaecological Cancer Centre, Royal Hospital for Women, Randwick, Sydney NSW 2031, Australia; 3 Faculty of Medicine, University of NSW, Randwick, Sydney NSW 2031, Australia

**Keywords:** ovarian cancer, CCBE1, tumour suppressor

## Abstract

**Background:**

*Collagen and calcium-binding EGF domains 1 (CCBE1)* is an uncharacterised gene that has down-regulated expression in breast cancer. As *CCBE1* maps to 18q21.32, a region frequently exhibiting loss of heterozygosity in ovarian cancer, the aim of this study was to determine the expression and function of CCBE1 in ovarian cancer.

**Methods:**

Expression and methylation patterns of *CCBE1* were determined in ovarian cancer cell lines and primary tumours. CCBE1 contains collagen repeats and an aspartic acid/asparagine hydroxylation/EGF-like domain, suggesting a function in extracellular matrix remodelling and migration, which was determined using small-interfering RNA (siRNA)-mediated knockdown and over-expression of *CCBE1* in cell lines.

**Results:**

CCBE1 is expressed in normal ovary, but is reduced in ovarian cancer cell lines and primary carcinomas. Pharmacological demethylation/deacetylation in ovarian cancer cell lines re-induced CCBE1 expression, indicating that epigenetic mechanisms contribute to its silencing in cancer. *CCBE1* promoter hypermethylation was detected in 6/11 (55%) ovarian cancer cell lines and 38/81 (41%) ovarian carcinomas. siRNA-mediated knockdown of CCBE1 in ovarian cancer cell lines enhanced their migration; conversely, re-expression of CCBE1 reduced migration and survival. Hence, loss of *CCBE1* expression may promote ovarian carcinogenesis by enhancing migration and cell survival.

**Conclusions:**

These data suggest that *CCBE1* is a new candidate tumour suppressor in ovarian cancer.

Ovarian cancer is the fifth leading cause of cancer death in women and has the highest mortality rate of the gynaecological cancers (Jemal *et al*, 2008). Most patients with ovarian carcinomas are diagnosed when their disease has disseminated throughout the peritoneal cavity and have a 5-year overall survival rate of only ∼20% (Barnholtz-Sloan *et al*, 2003). The identification of genes and pathways that contribute to the rapid metastasis of ovarian carcinomas may identify novel targets for therapeutic approaches to improve its poor prognosis.

*Collagen and calcium-binding EGF domains 1 (CCBE1)* is an earlier uncharacterised gene of unknown function that has recently been reported to be down-regulated in primary breast carcinomas as compared with matched normal breast tissue (Yamamoto and Yamamoto, 2007). *CCBE1* maps to 18q21.32, a common region of loss of heterozygosity (LOH) in ovarian cancer (Takakura *et al*, 1999; Lambros *et al*, 2005) that is associated with malignant progression of ovarian cancer (Hauptmann *et al*, 2002) and high tumour grade and poor survival (Lassus *et al*, 2001). *CCBE1* resides distal to the tumour suppressor genes (TSG) *DCC*, *SMAD2* and *SMAD4*, in a location suggested by microsatellite marker mapping to contain at least one additional as yet unidentified ovarian cancer TSG (Lassus *et al*, 2001). Although its function has not been described, *CCBE1* encodes a protein of 44 kDa that contains a number of recognised structural domains, including an EGF-like domain that incorporates an aspartic acid/asparagine (Asp/Asn) hydroxylation site. These domains are a feature of proteins with a demonstrated role in functions linked to metastasis, particularly cellular motility, including Notch family members and extracellular matrix molecules and is mediated by hydroxylation by the enzyme aspartyl *β*-hydroxylase (BAH/AAH) (Engel, 1989; Gronke *et al*, 1989; Lieber *et al*, 1992; Monkovic *et al*, 1992; Sepe *et al*, 2002; Maeda *et al*, 2003; de la Monte *et al*, 2006). Although its tissue distribution is not well described, CCBE1 is highly expressed in normal ovary as compared with other tissue types (Shyamsundar *et al*, 2005), and shares amino-acid sequence homology with the *Drosophila* vitellogenin receptor, an ovarian-specific, oestrogen-regulated gene, which is important in the development of the normal ovary (Schonbaum *et al*, 1995, 2000).

Analysis of oligonucleotide probesets correlating to *CCBE1* in our earlier reported genome-wide transcriptional profiling study of primary ovarian carcinomas (Heinzelmann-Schwarz *et al*, 2004, 2006) identified that CCBE1 expression was highly down-regulated in ovarian carcinomas of all histological subtypes as compared with normal ovary. A search of publicly available similar studies using the Oncomine database (http://www.oncomine.org) similarly showed that loss of CCBE1 expression in ovarian carcinoma as compared with normal ovarian surface epithelium (NOSE), the proposed site of origin of ovarian carcinomas, was evident in a study performed by Lu *et al* (2004).

The aims of this study were to determine whether CCBE1 expression is lost in ovarian cancer including primary carcinomas, and, given a putative functional link to migration, to determine whether CCBE1 loss affects cancer cell migration and survival. Furthermore, given that a dense region of CG dinucleotides (CpG island) spans its promoter region, a common feature of TSG (Jones and Laird, 1999), we also determined whether loss of *CCBE1* expression in ovarian cancer is related to aberrant epigenetic mechanisms, particularly hypermethylation of its promoter. These data identify *CCBE1* as a new candidate TSG in ovarian cancer.

## Materials and methods

### Cell lines and primary tumour samples

Ovarian (A2780, IGROV1, OV-90, CaOV3, TOV112D, TOV21G, SKOV-3, EFO27, OVCA420, CoLo316, OVCAR3) and breast cancer cell lines (T-47D, BT549, MCF-7, MDA-MB-453, MDA-MB-157, MDA-MB-231, MDA-MB-361, BT474, SK-BR3, HS578T, ZR75-1, BT483) were maintained under recommended growth conditions. Immortalised ovarian epithelial cell lines HOSE6.3 and 17.1 were kindly provided by Tsao *et al* (1995) and were maintained in MCDB 105 (Sigma Aldrich, Castle Hill, Australia) and Medium 199 (1 : 1; Invitrogen Life Technologies, Mt Waverley, Australia) containing 10% FBS. Normal breast cell lines (HMEC184, MCF10A, HMEC219.4) were maintained in HuMEC ready medium (Invitrogen) containing 1% HuMEC supplement and 50 mg l^–1^ bovine pituitary extract. The T-47DmEcoR cell line was made by stable transfection of the murine Eco receptor (EcoR) into T-47D cells to allow retroviral infection (Musgrove *et al*, 2001).

Primary tumour samples from 81 ovarian cancer patients were obtained from those surgically treated in the Gynaecological Cancer Centre, Royal Hospital for Women, Sydney, after informed consent. The clinical and pathological data on each patient were collected and are shown in Supplementary Table 1. Tissue samples for DNA/RNA extraction were collected immediately after surgical resection, snap frozen in liquid nitrogen and stored at −80°C. Representative sections were examined to confirm the histopathological diagnosis and ensure that the percentage of tumour content for each specimen was at least 80%. Archival tissue samples were processed in formalin and paraffin embedded according to standard pathological procedures. NOSE scrapings collected from the ovarian surface (*n*=18) were obtained from patients undergoing surgery for benign gynaecological conditions or non-ovarian gynaecological cancers. NOSE samples were collected into RNAprotect (Qiagen, Hilden, Germany) and stored at 4°C until processing . All experimental procedures were approved by the Human Research Ethics Committee of the Sydney South East Area Hospital Service, Northern Section (00/115).

### Antibodies

A rabbit polyclonal anti-human CCBE1 antibody was generated by immunising rabbits with the peptide (CDHPRRTETRDLRAPRDFYP) located at the C-terminal end of CCBE1 (Invitrogen). Post-immunisation sera from two rabbits was affinity purified, and specificity for CCBE1 confirmed using western blotting against the immunising peptide and against recombinant CCBE1 generated by retroviral over-expression as detailed below. A monoclonal antibody specific for human GAPDH was purchased from Ambion (Austin, TX, USA); horseradish peroxidase (HRP)-conjugated anti-rabbit IgG and anti-mouse IgG antibodies were purchased from Amersham Biosciences (Little Chalfon, UK).

### Real-time PCR

RNA was extracted from cell lines and primary tissue using the RNAeasy mini kit (Qiagen). Total RNA (1 *μ*g) was treated with DNase1 (Ambion), then reversed transcribed to cDNA using oligo-dT primers (Promega, Madison, WI, USA). Real-time quantitative PCR was performed on an ABI Prism HT7900 sequence detection system (Applied Biosystems, Foster City, CA, USA) CCBE1 or GAPDH TaqMan primers (Applied Biosystems). CCBE1 expression was calculated by using the difference-in-threshold-cycle (Δ*C*_t_) parameter normalising to GAPDH (internal reference gene) in each sample, then expressed relative to a reference sample that expressed CCBE1.

### Western blotting

Whole cell lysates from ovarian cell lines were prepared in modified RIPA supplemented with protease inhibitors. Protein concentration was assessed by the Bradford Assay (Bio-Rad, Hercules, CA, USA). Denaturing SDS–PAGE and western blotting were performed according to standard protocols. CCBE1 expression was determined by overnight incubation in a 1 : 1000 dilution of affinity-purified rabbit polyclonal CCBE1 antibody at 4°C. Detection of GAPDH expression (1 : 10 000) was used as a loading control. After washing, membranes were incubated with HRP-conjugated rabbit (CCBE1) or mouse (GAPDH) anti-IgG antibody (1 : 10 000; Amersham Biosciences) followed by white lightening ECL (PerkinElmer LAS, Boston, MA, USA) signal detection and densitometry.

### *In situ* hybridisation

DIG-labelled RNA probes were made by amplifying target DNA from normal ovarian cDNA using the Expand High Fidelity PCR System (Roche, Mannheim, Germany) and the following primers: F3, CACAATAGACCATAACTCCT and R3, AAACGCCATTCAATTCTCTC. A T7 promoter adapter was ligated to the ends of both the sense and antisense PCR products (Lig’n Scribe, Ambion). *In situ* hybridisation (ISH) using 0.1 ng *μ*l^–1^ of antisense or sense (control) DIG-labelled probes (DIG RNA Labelling Mix, Roche) was performed on formalin-fixed, paraffin-embedded tissue sections as described earlier (Heinzelmann-Schwarz *et al*, 2004), using a Ventana-DISCOVERY system. A 1 : 500 dilution of anti-DIG biotin-labelled monoclonal antibody (D1-22; Sigma Aldrich) for 30 min at 37°C was used for detection of probe signal.

### Methylation assays

Genomic DNA was isolated from cell lines and primary tissue using the QIAmp DNA Mini kit (Qiagen). Cancer DNA was bisulphite converted in 96-well plates using the 96 EZ DNA Gold kit (Zymo Research Corps, Orange, CA, USA). NOSE DNA was bisulphite converted as described (Clark *et al*, 2006). A total of 40 ng of bisulphite-converted DNA was PCR amplified using the following primers and probe for the *GSTPi* gene as a positive control for complete bisulphite conversion as described earlier (Rand *et al*, 2005). To determine methylation in *CCBE1*, bisulphite-converted DNA was first PCR amplified using 250 nM of the following primers F1: 5′-AGGGAAGTGTCGTTTAGGATAGTTGAG-3′ and R1 5′-TAAAAAAAAACCGAAAACTTCCCTAATAATA-3′ (321 bp). The conditions for amplification were 95°C for 4 min, then 5 cycles of 95°C for 45 s, 58.5°C for 90 s and 72°C for 2 min, and then 45 cycles of 95°C for 45 s, 58.5°C for 30 s and 72°C for 30 s. The resulting PCR products were purified and directly sequenced using the BigDye Terminator v3.1 sequencing kit (PerkinElmer) on an ABI Prism 3100 Genetic Analyser (Hitachi; Applied Biosystems, Scores, VIC, Australia). The methylation pattern obtained by sequencing was used to design methylation-specific primers and probe using Primer Express software (Applied Biosystems) as follows: CCBE1-MSPF1 5′-GGAGGATCGTTTGTATTTCGCGAGTC-3′ and CCBE1-MSPR2 5′-AACCTACAAAAAAAAACCGAAAAACGACG-3′, probe FAM-5′-CGCGTATTAAGTAGGAGTTCGTTT-3′-MGB (110 bp). qMSP assays were performed in triplicate using Platinum PCR buffer (Invitrogen), 200 nM of each primer, 150 *μ*M probe, 0.3 U of Platinum Taq polymerase (Invitrogen) and 40 ng bisulphite-converted DNA, with amplification conditions 95°C for 2 min, and then 45 cycles of 95°C for 15 s and 60°C for 60 s in an ABI HT7000 (Applied Biosystems). Bisulphite-treated CpGenome universal methylated DNA (Chemicon International, Temecula, CA, USA) was used as a positive control, and a bisulphite-treated clone isolated from unmethylated control DNA (Roche) that was fully unmethylated in the *CCBE1* promoter region was used as a negative control. To confirm the qMSP results, PCR products were cloned into pGEM T Easy Vector (Promega) and transformed into *Escherichia. coli* using standard procedures. Plasmid DNA was isolated from randomly selected colonies (12) and directly sequenced.

### Treatment of cell lines with epigenetic inhibitors

For demethylation studies, HOSE6.3, A2780, CaOV3, OV90, SKOV3 and TOV21G cells were treated with 5 *μ*M 5-aza-2′-deoxycytidine (5-AZA; Sigma Aldrich) 24 h after plating in 10 cm dishes. After 48 h, fresh medium containing 100 nM trichostatin A (TSA; Sigma Aldrich) was added for a further 24 h. Control cells were untreated. Cells were washed in PBS and harvested in RLT buffer (Qiagen) before RNA extraction.

### Knockdown of CCBE1 expression using small-interfering RNA

CoLo316 cells (4.65 × 10^5^) were seeded in 10 cm dishes 16 h before transfection, then transfected with 5 nM of On Target Plus small-interfering RNA (siRNA) against CCBE1 or a control non-targeting RISC-free (siGLO) siRNA (Dharmacon, Lafayette, IN, USA) for 6 h using Lipofectamine 2000 (Invitrogen). At 72 h post-transfection, cells were washed in PBS before RNA or protein lysate extraction.

### Retroviral-mediated over-expression of CCBE1

A cDNA encoding full-length *CCBE1* was amplified from HOSE 6.3 cells using PfuUltra Hotstart High-Fidelity DNA polymerase (Agilent) using the following primers: 5′-G GGG ACA AGT TTG TAC AAA AAA GCA GGC TTC ACC ATG GTG CCG CCG CCT CCG AGC CGG-3′ and 5′-GGG GAC CAC TTT GTA CAA GAA AGC TGG GTC TGG GTA GAA GTC TCT GGG GGC T-3′ and cloned into the Gateway donor vector pDONR (Invitrogen) and sequenced. *CCBE1* was subcloned into pMCVS_IRES_GFP (pMIG) with the addition of a C-terminal V5 tag, then transfected into Phoenix cells using FuGene 6 (Roche). Replication-deficient retroviruses were collected from the culture medium after 48 h and applied to T-47DmEcoR cells seeded at a density of 1.2 × 10^6^ in 10 cm dishes 16 h earlier. T-47DmEcoR cells were grown to confluence in complete growth media supplemented with 30 *μ*g ml^–1^ polybrene (Sigma Aldrich). Cells were then harvested and sorted according to low and high GFP expression using a FACS Vantage SE with FACS DiVa option and DiVa 4.1.2 software (Becton Dickinson, Franklin Lakes, NJ, USA), before re-passaging. Total RNA and protein lysates were extracted for quantification of CCBE1 expression as described above.

### Migration assays

CoLo316 cells (1.75 × 10^4^) or FACS-sorted vector only/GFP- and CCBE1/GFP-expressing T-47DmEcoR cells (6 × 10^4^) were added to the upper chamber of 8 *μ*M porosity transwells (BD) coated on the lower side with 3 mg ml^–1^ collagen I (Sigma Aldrich). Growth medium was added to the bottom well and cells were allowed to migrate overnight in a 37°C humidified CO_2_ incubator. Each assay was performed in duplicate transwells, and at least two independent experiments were performed. Non-migrating cells were removed from the upper chamber with a cotton swab and the membrane was stained with DiffQuik (Lab Aids, Ronkonkoma, NY, USA). Migration was quantified by counting cells per 30 fields of view using × 20 magnification. The mean number of cells per field of view was calculated and averaged between duplicate transwells. The results were expressed as the relative percentage of migrating cells as compared with vector control/GFP high cells.

### Colony-forming assays

FACS-sorted vector/GFP- and CCBE1/GFP-expressing T-47DmEcoR cells (1 × 10^3^ cells per well) were plated into six-well plates and incubated for 8 days to allow colonies to form. Colonies were stained with DiffQuik (Lab Aids), then scanned and quantified (ChemiDoc XRS and Quantity One 4.5.1 software; Bio-Rad). Average colony density was calculated and results expressed as the relative percentage of colonies as compared with vector control/GFP high cells.

### Statistical analyses

Statistical analysis of differences between means was performed using Wilcoxon/Mann Whitney *U* non-parametric tests or ANOVA as appropriate. Correlations between *CCBE1* methylation and clinical and pathological variables were determined using the Mann–Whitney *U*-test. Association of *CCBE1* expression or methylation with recurrence-free survival was evaluated by Kaplan–Meier analysis. Patients that had not suffered an event (recurrence of ovarian cancer) or who were lost to follow up were censored. Recurrence-free survival was only evaluated in patients who exhibited a complete response to treatment (defined as no clinical, radiological or tumour marker evidence of disease for 3 months post-treatment), and was measured from the date of diagnosis to the date of last follow-up or to disease recurrence (defined as either the reappearance of clinical symptoms by clinical examination or radiological investigation, or a rising serum CA 125 level >35 U ml^–1^). All statistical analyses were performed using Statview 4.5 (Abacus Systems, Berkeley, CA, USA) and *P*<0.05 was considered statistically significant.

## Results

### *CCBE1* is frequently down-regulated in ovarian and breast cancer cell lines and primary ovarian carcinomas

**Figure 1:**
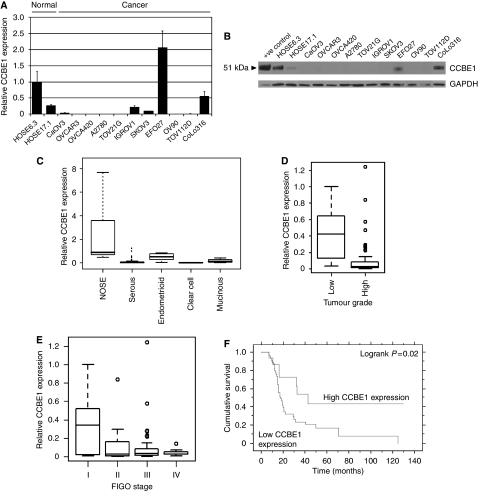
CCBE1 expression is lost or reduced in ovarian and breast cancer. (**A**) Real-time quantitative PCR showing CCBE1 mRNA expression in a panel of ovarian cancer cell lines as compared with immortalised HOSE 6.3 cells. CCBE1 mRNA was normalised to GAPDH mRNA expression levels. (**B**) Western blot analysis showing CCBE1 expression (51 kDa) in ovarian cancer cell lines as compared with HOSE 6.3 cells. A positive control protein lysate was extracted from IGROV1 cells transiently transfected with V5-tagged CCBE1. (**C**) Box plot of CCBE1 mRNA expression in NOSE (*n*=14) as compared with serous (*n*=64), endometrioid (*n*=5), clear cell (*n*=5) and mucinous (*n*=4) ovarian carcinomas. (**D**) Box plot of CCBE1 mRNA expression in low (grade 1; *n*=7) and high (grade 2,3, *n*=71) grade ovarian carcinomas. (**E**) Box plot of CCBE1 expression in FIGO stage I (*n*=10), stage II (*n*=10), stage III (*n*=51) and stage IV (*n*=7) ovarian carcinomas. (**F**) Kaplan–Meier survival curve for relapse-free survival time stratified by CCBE1 expression in all ovarian carcinomas.

CCBE1 mRNA expression was down-regulated or undetectable in 8/11 (73%) ovarian cancer cell lines as compared with normal (immortalised) ovarian surface epithelial cells, as determined using quantitative TaqMan PCR (qPCR; Figure 1A). To confirm earlier reported results in primary breast carcinomas, we also examined expression of CCBE1 in breast cancer cell lines, in which 8/12 (67%) showed loss of expression as compared with normal breast cell lines (Supplementary Figure 1). Loss of CCBE1 expression in ovarian cancer cell lines was further confirmed by western blotting using a polyclonal antibody raised against CCBE1 (Figure 1B). There was a strong correlation between CCBE1 mRNA and protein levels: CCBE1 protein expression was only seen in HOSE 6.3 and 17.1 cells and in the ovarian cancer cell lines EFO27 and CoLo316 that retained the highest level of CCBE1 mRNA and protein.

**Figure 2:**
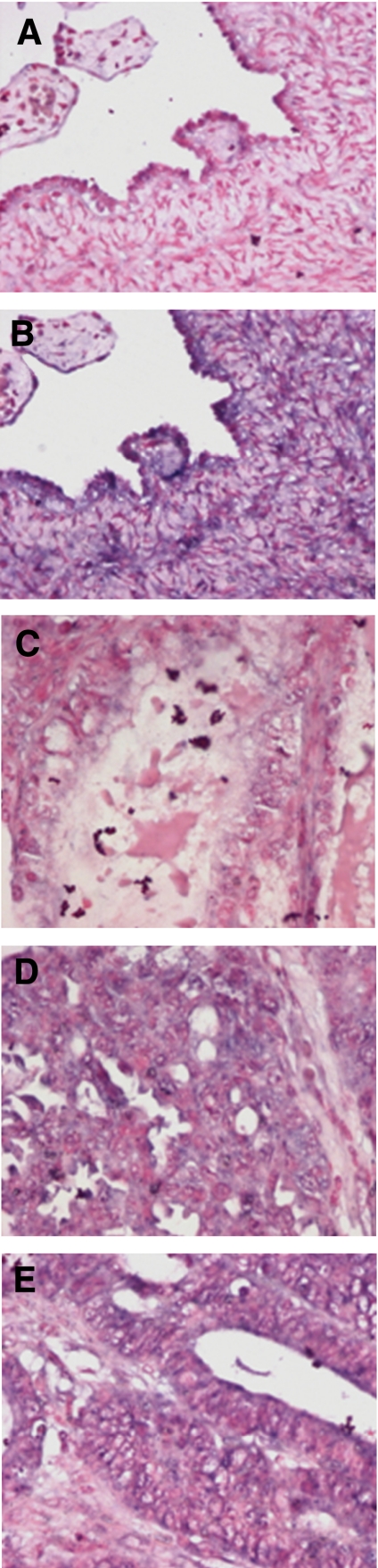
Representative ISH for CCBE1 mRNA in normal ovary using (**A**) sense negative control and (**B**) antisense probes, respectively; (**C**) mucinous ovarian carcinoma; (**D**) serous ovarian carcinoma; (**E**) endometrioid ovarian carcinoma, all with antisense probe (magnification × 20).

qPCR analysis of 78 primary ovarian carcinomas and 14 NOSE cell brushings determined that CCBE1 was significantly down-regulated in ovarian cancers of all histological subtypes as compared with NOSE (serous, mucinous, clear cell *P*<0.001; endometrioid *P*<0.05; Figure 1C). CCBE1 expression was also significantly lower in high grade as compared with low grade carcinomas (*P*=0.002; Figure 1D), but did not differ between FIGO stages (*P*=0.3; Figure 1E), suggesting that loss of expression occurs early in carcinogenesis. Low CCBE1 expression was also significantly associated with earlier disease recurrence (logrank *P*=0.02; Figure 1F). As there is no anti-CCBE1 antibody suitable for immunohistochemistry, we examined CCBE1 expression in a small sample of primary carcinomas using ISH, which confirmed that CCBE1 mRNA was highly expressed in both NOSE and in normal ovarian stroma, as predicted by transcriptional profiling studies (Figure 2). In addition, ISH provided visual evidence that CCBE1 mRNA expression is at least reduced in mucinous, serous, and endometrioid ovarian carcinomas as compared with normal ovary. Together, these studies indicate that CCBE1 is normally expressed in the ovary, but is frequently down-regulated in ovarian and breast cancer.

### Methylation of *CCBE1* contributes to down-regulated expression in ovarian cancer cell lines and primary ovarian carcinomas

**Figure 3:**
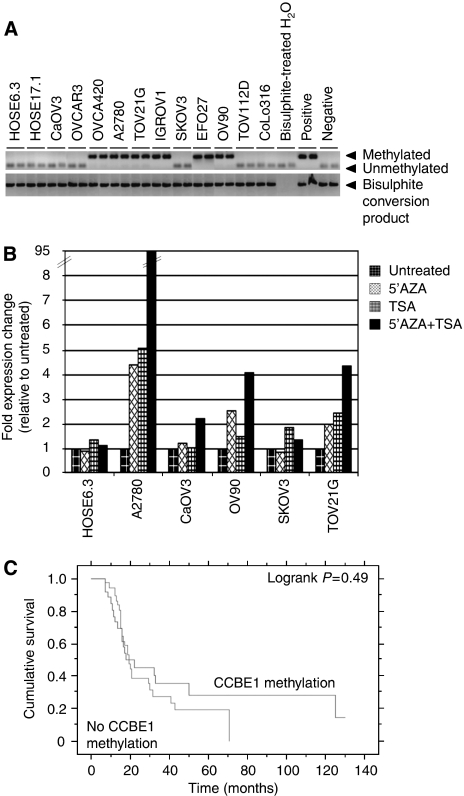
*CCBE1* is methylated in ovarian cancer cell lines and primary carcinomas. (**A**) Methylation-specific PCR analysis of bisulphite-treated DNA from HOSE 6.3 and ovarian cancer cell lines. Positive control is bisulphite-treated CpGenome universally methylated DNA; negative control is bisulphite-treated genomic unmethylated DNA. (**B**) Normalised expression of CCBE1 mRNA in HOSE 6.3 and five ovarian cancer cell lines after treatment with 5-AZA, TSA or in combination, as compared with untreated cells. (**C**) Kaplan–Meier survival curve for relapse-free survival time stratified by *CCBE1* methylation status in all ovarian carcinomas.

Bioinformatic analysis of the predicted *CCBE1* promoter using Genome Browser (UCSC) identified a CpG island spanning the transcriptional start site (nt −411 to 875), suggesting that *CCBE1* could be susceptible to methylation-associated transcriptional silencing. As aberrant DNA methylation is a well-recognised mechanism of gene silencing in cancer (Jones and Laird, 1999; Herman and Baylin, 2003), we next determined whether the putative promoter region of *CCBE1* was methylated in ovarian cancer. Primer and probe sequences for a quantitative methylation-specific PCR (qMSP) assay were designed upstream of the transcriptional start site and qMSP performed on bisulphite-treated DNA samples from ovarian cell lines. In total, 10 CpG dinucleotides were interrogated by the primers and probe. The qMSP for CCBE1 had a sensitivity of 25 pg and specificity of 1 : 1000 (1 methylated allele in a background of 1000 unmethylated alleles), as determined using limiting dilution of methylated:unmethylated DNA. Methylated DNA was detected in 6/11 (55%) of ovarian cancer cell lines, but not in HOSE 6.3 or 17.1 cells (Figure 3A). Direct sequencing of two cell lines (IGROV1 and OVCA420) confirmed their methylation status (Supplementary Figure 2A). Five out of six cell lines that exhibited promoter methylation also displayed a reduction in or total loss of CCBE1 mRNA expression (Figure 1A).

To determine whether CpG island methylation directly mediates *CCBE1* silencing, we compared CCBE1-expression levels in ovarian cancer cell lines before and after treatment with the methyltransferase inhibitor 5-aza-2′-deoxycytidine (5-AZA) with or without the histone deacetylase inhibitor TSA. No difference in CCBE1 mRNA expression was observed in HOSE 6.3, CaOV3 and SKOV3 cell lines, which were not methylated in the promoter region of *CCBE1*. However, there was an increase in CCBE1 mRNA in the *CCBE1* methylated cell lines A2780, OV90 and TOV21G cell lines after 5-AZA treatment as compared with untreated cells (Figure 3B), supporting hypermethylation of the *CCBE1* promoter as one mechanism mediating loss of expression. Moreover, re-expression of CCBE1 mRNA after treatment with combined 5-AZA and TSA suggests that deacetylation of histones also affects expression of CCBE1 in cancer.

To determine whether hypermethylation of the *CCBE1* promoter occurs in primary ovarian cancers, qMSP was performed on bisulphite-treated genomic DNA extracted from fresh frozen tissue from 81 ovarian carcinomas, 5 NOSE and microdissected ovarian stroma from normal ovaries (*n*=4). Approximately 41% (33/81) of ovarian carcinomas exhibited methylation in the *CCBE1* promoter as compared with 20% (1/5) NOSE and 0% (0/4) normal ovarian stroma samples (Table 1). Two primary ovarian tumours (1179 and 1398) were sequenced to validate their positive methylation status (Supplementary Figure 2B). However, there was neither association of methylation with histological subtype, FIGO stage, tumour grade, age at diagnosis, pre-operative CA125 levels or ascites volume (data not shown), nor any association between *CCBE1* methylation and earlier disease recurrence (Figure 3C) in women with ovarian cancer.

### Down-regulation of CCBE1 in ovarian cancer cells promotes cell migration

**Figure 4:**
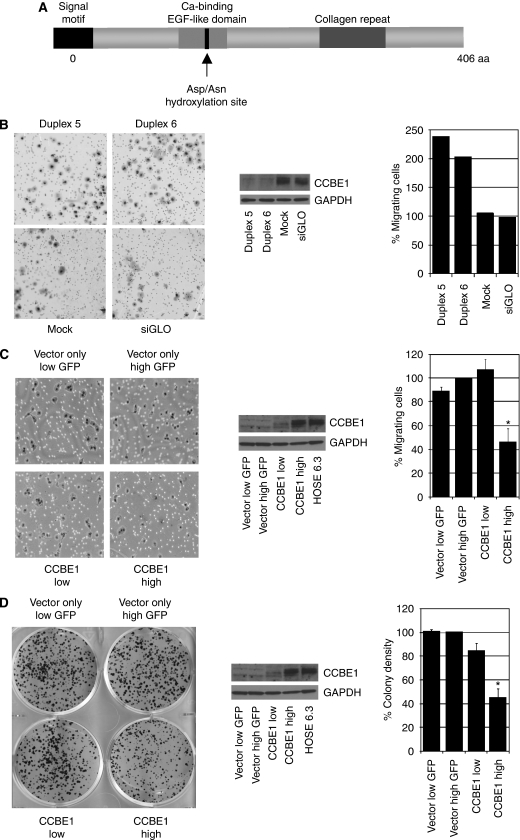
Modulation of CCBE1 levels affects cancer cell line behaviour. (**A**) Cartoon depicting the structural domains of CCBE1, which predict a function in cross-talk between the ECM and NOSE. (**B**) Knockdown of CCBE1 expression by siRNA in CoLo316 cells increases cell migration. The graph shows the number of migrating cells expressed as a percentage of siGLO scrambled control from two independent experiments. Expression of CCBE1 72 h post-transfection was determined by western blotting. (**C**) Over-expression of CCBE1 in T-47D cells decreases cell migration. The graph shows the number of migrating cells expressed as a percentage of vector high GFP controls from three independent experiments (^*^P<0.013, *n*=5). CCBE1 over-expression was confirmed by western blotting and was comparable with endogenous expression of CCBE1 in HOSE6.3 cells. (**D**) Over-expression of CCBE1 in T-47D cells decreases their colony-forming ability. The graph shows colony density expressed as a percentage of vector control cells expressing high levels of GFP from five independent experiments (^*^P<0.01, *n*=15). All images show representative fields of view (magnification × 20).

The frequent and early silencing of CCBE1 expression in ovarian cancer suggests that CCBE1 may have a function in its pathogenesis. Although the function of CCBE1 is not yet characterised, its predicted structural domains provide clues to its potential function (Figure 4A): CCBE1 contains an N-terminal signalling domain, suggesting that it may be a secreted protein (Clark *et al*, 2003); two collagen repeats (GXY motif), identifying it as a non-collagen member of the collagen superfamily and a calcium-binding EGF-like domain that incorporates the consensus aspartic acid/asparagine (Asp/Asn) sequence for hydroxylation by the aspartyl (asparaginyl) *β*-hydroxylase. This sequence has been identified in ∼30 known proteins including signalling molecules such as Notch and Notch homologs, and ECM molecules such as laminin and tenascin, which have showed functions in cell migration or adhesion, dominant processes in metastasis (Engel, 1989; Gronke *et al*, 1989; Lieber *et al*, 1992; Monkovic *et al*, 1992; Sepe *et al*, 2002; Maeda *et al*, 2003; de la Monte *et al*, 2006). As an initial investigation to identify a putative functional role for CCBE1, we, therefore, investigated whether CCBE1 has a function in cancer cell migration. We used transient transfection of CCBE1-specific siRNAs to knock down expression of CCBE1 in the ovarian cancer cell line CoLo316, which had the highest endogenous protein expression of CCBE1 (Figure 1B). Using two independent siRNAs targeting CCBE1 mRNA (duplex 5 and 6), we saw an almost total loss of CCBE1 expression at 72 h post-transfection (Figure 4B). The specificity of siRNA knockdown was confirmed by using a scrambled CCBE1 sequence control that did not cause loss of CCBE1 mRNA (data not shown). The effect of CCBE1 loss on cell migration was investigated using transwell assays, in which CCBE1 siRNA-depleted cells were allowed to migrate towards collagen I, one of the most abundant glycoproteins in the ECM. CCBE1 depletion caused a reproducible 100% increase in the number of cells that migrated through the transwells relative to mock-transfected cells and to cells transfected with a control siRNA (siGLO) (Figure 4B). Thus, loss of CCBE1 expression enhances migration of ovarian cancer cells *in vitro*.

### Overexpression of CCBE1 inhibits cell migration in breast cancer cells

To further expand this observation, we next wanted to determine whether re-expression of CCBE1 through a plasmid expression construct would inhibit migration of ovarian cancer cells. Despite several attempts, we were unable to isolate lines that retained stable expression, suggesting that high expression of CCBE1 was incompatible with growth or survival (data not shown). This observation agrees with our expression results, in which only two of a panel of 11 ovarian cancer cell lines tested showed significantly detectable levels of CCBE1 (Figure 1A). Given that CCBE1 expression is also reduced in breast cancer, we alternatively infected T-47D breast cancer cells expressing the murine ecotropic receptor (T-47DmEcoR) with a retroviral vector-expressing full-length CCBE1. T-47D cells express undetectable endogenous levels of CCBE1 (Supplementary Figure 1). The generated expression construct co-expressed GFP through an internal ribosome entry site (IRES) linked to CCBE1 and thus allowed transfected cell populations to be FACS sorted for GFP expression corresponding to CCBE1 expression. Western blotting confirmed low CCBE1 expression in low GFP-expressing cells and high CCBE1 expression in the GFP high cell population, which was comparable with the level of CCBE1 in HOSE 6.3 cells (Figure 4C). We next tested the ability of the T-47DmEcoR/CCBE1-expressing cells or corresponding vector only control cells to migrate in transwell assays. Migration towards collagen I was impaired approximately two-fold in the high CCBE1-expressing cells compared with their control-transfected counterparts (*P*=0.013; Figure 4C). This result was reproducible in two independently generated sets of CCBE1/GFP-expressing cells (data not shown). Thus, CCBE1 expression is inversely associated with migration of breast cancer cells *in vitro*.

### Re-expression of CCBE1 reduces colony-forming ability in breast cancer cells

As T-47D cells readily form colonies when sparsely seeded on plastic, we additionally examined whether expression of CCBE1 in T-47DmEcoR cells had a suppressive effect on the colony-forming ability of cancer cells. Expression of CCBE1 significantly inhibited colony formation in comparison with vector controls (*P*<0.05; Figure 4D). Thus, expression of CCBE1 is also inversely correlated with tumour cell survival. As the ovarian cancer cell lines, which express endogenous CCBE1, do not form distinct colonies, we were unable to assess whether siRNA-mediated knockdown of CCBE1 would conversely promote survival.

## Discussion

We report here that loss of CCBE1 expression is a common event in ovarian cancer, is associated with poor patient outcome and that its loss enhances cancer cell migration and survival, which together suggest that *CCBE1* is a new candidate TSG. As earlier reported in primary breast cancers (Yamamoto and Yamamoto, 2007), we found that CCBE1 expression was down-regulated in the majority of ovarian cancer cell lines tested and in primary ovarian carcinomas as compared with NOSE. Loss of CCBE1 expression was significantly associated with higher grade ovarian carcinomas and likely occurs early in ovarian carcinogenesis, with stage I carcinomas expressing lower levels of CCBE1 than NOSE, one predicted cellular origin of ovarian carcinomas. Moreover, loss of CCBE1 expression was associated with earlier disease recurrence, indicative of a functional role for CCBE1, or the cellular pathways in which it is involved, in ovarian carcinogenesis. These data remain to be confirmed at later follow-up and additionally investigated in larger retrospective studies.

In addition to genetic alterations, epigenetic silencing of tumour suppressors is well documented in cancer (Jones and Laird, 1999; Herman and Baylin, 2003) and is likely one of the earliest molecular changes in carcinogenesis (Jones and Baylin, 2002). Epigenetic inactivation can account for silencing of the remaining allele after loss of the first allele due to genetic mechanisms such as deletion or mutation, or can account for inactivation and silencing of both alleles (Knudson, 2001; Jones and Baylin, 2002). Like other cancers, many candidate TSG are reported to be regulated by epigenetic mechanisms in ovarian cancer (for review see, Barton *et al*, 2008). This study identifies that CCBE1 expression in ovarian cancer is at least in part regulated by epigenetic mechanisms, in particular promoter hypermethylation. However, we did not find any association between *CCBE1* methylation and clinicopathological parameters, including FIGO stage, tumour grade or age at diagnosis, nor any evidence that CCBE1 methylation could predict earlier disease recurrence. Moreover, expression of *CCBE1* was not exclusively correlated with promoter methylation, suggesting that additional mechanisms contribute to its silencing in ovarian cancer. Chromatin remodelling also likely has a function, given that treatment with a de-acetylating agent also increased CCBE1 expression in the ovarian cancer cell lines. Furthermore, we identified *CCBE1* as a candidate TSG at least partly due to its genomic location at 18q21, a region of known LOH in ovarian cancer (Takakura *et al*, 1999; Lambros *et al*, 2005). The microsatellite marker D18S64, located in the 3′ flanking region of *CCBE1*, shows 58% allelic loss in ovarian cancer (Lassus *et al*, 2001), implying that LOH may also be an important mechanism involved in loss of CCBE1 expression; however, this remains to be determined.

Little is known about the function(s) of CCBE1 and, therefore, how its loss may potentiate carcinogenesis. However, its structural motifs including the presence of collagen repeats and an Asp/Asn hydroxylation motif, together with its expression in both the surface epithelium and stroma of the ovary, are suggestive of a function in cross-talk between the surface epithelial cells and the ECM that affects cellular migration. In the normal ovary, ECM remodelling occurs after ovulatory rupture, in which the ovarian surface epithelial cells convert to a mesenchymal, fibroblast-like phenotype (epithelial-mesenchymal transition), resulting in increased motility, proliferation and capacity to modify the ECM, allowing repair of the surface epithelial layer (Auersperg *et al*, 2001). ECM remodelling also contributes to cancer metastasis, in which it increases capacity of cells to migrate and survive (Egeblad and Werb, 2002; van Kempen *et al*, 2003). Our results show that cells that have lost CCBE1 expression have an increased migratory potential and survival advantage, further evidence of a function in cell migration. Loss of CCBE1 expression may, therefore, modulate the migration of cancer cells from the ovary into the peritoneum, a particular characteristic of ovarian carcinomas. Further experiments detailing its cellular location, expression and function in normal and cancer cells will provide further insight into the function of CCBE1 and how it might contribute to carcinogenesis.

The only enzyme currently known to catalyse hydroxylation of Asp/Asn EGF-like domains is aspartyl *β*-hydroxylase (BAH/AAH), which is expressed in normal ovary, at least in mice (Dinchuk *et al*, 2000). Female BAH knockout mice have developmental abnormalities and decreased fertility, and are more susceptible to tumour formation (Dinchuk *et al*, 2002). If CCBE1 activity is modulated by hydroxylation of Asp/Asn residues by BAH, it could be predicted that silencing of CCBE1 may result in a similar tumour-promoting effect to that of BAH loss, although this remains to be determined. However, over-expression of BAH/AAH has been reported in a number of carcinomas (Lavaissiere *et al*, 1996; Cantarini *et al*, 2006) in which it mediates increased cellular motility/migration and invasion through hydroxylation of proteins containing the Asp/Asn consensus site (Engel, 1989; Gronke *et al*, 1989; Lieber *et al*, 1992; Monkovic *et al*, 1992; Sepe *et al*, 2002; Maeda *et al*, 2003; de la Monte *et al*, 2006), which is linked to cellular transformation (Ince *et al*, 2000). Relative expression levels of BAH in ovarian carcinomas have yet to be reported.

In summary, loss of CCBE1 expression, at least in part due to epigenetic silencing, is common in ovarian carcinomas and confers a migratory and survival advantage to cancer cells. *CCBE1* is thus a novel candidate TSG inactivated in early ovarian cancer development. Further studies including knockout mouse models will further determine its function in the regulation of ovarian cancer cell motility and survival, and hence its potential function in ovarian carcinogenesis and metastasis.

## Figures and Tables

**Table 1:** Methylation status of normal ovaries and primary ovarian carcinomas

	**CCBE1 methylation positive**
NOSE (*n*=5)	1 (20%)
Normal stroma (*n*=4)	0 (0%)
All cancers (*n*=81)	33 (40.7%)
Serous (*n*=67)	25 (37.3%)
Endometrioid (*n*=5)	2 (40%)
Clear cell (*n*=5)	3 (60%)
Mucinous (*n*=4)	3 (75%)

Abbreviations: CCBE1=collagen and calcium-binding EGF domains 1; NOSE=normal ovarian surface epithelium.
