# Supplementary material for: Collagen and calcium-binding EGF domains 1 is frequently inactivated in ovarian cancer by aberrant promoter hypermethylation and modulates cell migration and survival
Source: Br J Cancer. 2009 Nov 24;102(1):87–96. doi: 10.1038/sj.bjc.6605429 (PMC2813742; doi:10.1038/sj.bjc.6605429)
Supplement: Supplementary Table 1 [file 6605429x1.pdf]

	A	B	C	D	E	F	G	H	I	J	K	L	M	N	O	P	Q	R	S	T	U	V	W
1	Garvan ID	CCBE1 methy	Relative CCBE1 ex	Histotype	Diagnosis	Age Diagr	Stage	Grade	Residual Disease	Preop CA125	Response	Earliest Relapse	Relapse Time	Survival Time	Status	Cause Death	Menstrual	Adj Chemo 1	Adj Chemo 2	Adj Chemo 3	Ascites	Ascites Vol	
2	1022	N	0.001	Serous	Ov Ca	56.8	2	3	0	511	Complete response	70.9	70.9	73.1	Alive		Post	Carboplatinum	Taxol		Unknown		
3	1024	N	0.033	Serous	Ov Ca	64.7	3	1	1.5	756	Complete response		3	3	LFU		Post	Carboplatinum	Taxol		Yes		
4	1025	N	0.025	Serous	Ov Ca	44.7	3	2	>2	4570	Partial response			27.4	Deceased	Related to malignanc	Pre	Carboplatinum	Taxol		Yes	4000	
5	1029	N	0.147	Serous	Ov Ca	57.9	3	2	4	36100	Complete response	7.2	7.2	34.4	Deceased	Related to malignanc	Post	Carboplatinum	Taxol		No		
6	1031	Y	0.069	Serous	Ov Ca	47.1	4	3	10	5750	Partial response			18.5	Deceased	Related to malignanc	Pre	Carboplatinum	Taxol		No		
7	1033	Y	0.028	Serous	Ov Ca	59.9	3	3	<1	1354	Complete response		58.0	58.0	Alive		Post	Carboplatinum	Taxol		Yes	400	
8	1043	N	ND	Serous	Ov Ca	35.7	3	2	0.5	731	Complete response	18.6	18.6	50.3	Alive		Pre	Carboplatinum	Topotecan	Taxol	Yes		
9	1049	N	0.058	Serous	Ov Ca	80.4	3	2	0.5	263	Complete response	31.7	31.7	38.0	Deceased	Related to malignanc	Post	Carboplatinum			Yes	5000	
10	1076	N	0.006	Serous	Ov Ca	48.0	2	2	0	48	Complete response	29.9	29.9	64.8	Deceased	Unrelated to maligna	Pre	Carboplatinum	Taxol		No		
11	1099	N	0.139	Serous	Ov Ca	33.1	4	3	2	NK	No response			9.2	Deceased	Related to malignanc	Pre	Carboplatinum	Cyclophosphamide		Yes	200	
12	1101	N	0.008	Serous	Ov Ca	40.8	3	2	0.5	4300	Complete response	40.9	40.9	67.4	Alive		Pre	Carboplatinum			Yes	1000	
13	1110	N	0.008	Serous	Ov Ca	68.4	3	3	5	788	Partial response			21.5	Deceased	Related to malignanc	Post	Carboplatinum			Yes	2500	
14	1124	N	0.001	Serous	Ov Ca	59.9	3	2	0.5	2040	Complete response	20.8	20.8	52.9	Deceased	Related to malignanc	Post	Carboplatinum	Taxol		No		
15	1140	Y	0.011	Serous	Ov Ca	54.4	3	3	miliary	2041	Complete response	15.9	15.9	30.2	Alive		Post	Carboplatinum	Taxol		Yes	300	
16	1153	N	0.005	Serous	Ov Ca	56.9	3	3	1	304	Complete response	19.4	19.4	37.3	Alive		Post	Carboplatinum	Taxol		Yes	2000	
17	1166	N	0.031	Serous	Ov Ca	57.9	4	3	0.3	5110	Complete response	13.8	13.8	25.7	Deceased	Related to malignanc	Post	Cisplatinum	Cyclophospham		Yes		
18	1178	N	0.005	Serous	Ov Ca	59.3	3	3	<1	1316	Complete response	12.1	12.1	48.4	Alive		Post	Carboplatinum	Taxol		Yes	1500	
19	1179	Y	0.016	Serous	Ov Ca	74.3	3	3	miliary	190	Complete response	6.9	6.9	26.2	Deceased	Related to malignanc	Post	Carboplatinum			No		
20	1180	N	0.094	Serous	Ov Ca	49.0	3	3	miliary	1176	Complete response	18.7	18.7	28.0	Deceased	Related to malignanc	Post	Carboplatinum	Taxol	Taxatere	Yes	6000	
21	1182	Y	0.295	Serous	Ov Ca	77.3	2	3	0	57	Complete response		130.3	130.3	Alive		Post	Carboplatinum			No		
22	1203	N	0.574	Serous	Ov Ca	58.8	3	3	miliary	329	Partial response			19.8	Alive		Post	Carboplatinum			Yes	4000	
23	1205	N	0.473	Serous	Ov Ca	64.2	1	2	0	290	Complete response		46.7	46.7	Alive		Post	Carboplatinum	Taxol		No		
24	1207	N	0.022	Serous	Ov Ca	62.2	3	2	miliary	2430	Complete response	15.4	15.4	44.9	Alive		Post	Carboplatinum	Taxol		Yes	1000	
25	1234	N	0.282	Serous	Ov Ca	46.1	3	3	0	304	Complete response	42.5	42.5	46.5	Alive		Post	Carboplatinum	Taxol		Yes	100	
26	1239	N	0.020	Serous	Ov Ca	69.1	1	3	0	816	Complete response	15.3	15.3	36.7	Deceased	Related to malignanc	Post	Cisplatinum	Cyclophospham		Yes	100	
27	1332	Y	0.008	Serous	Ov Ca	58.9	3	3	<1	1120	Complete response	21.9	21.9	28.1	Deceased	Unknown	Post	Carboplatinum	Taxol	Doxil	Yes	200	
28	1350	Y (P)	0.112	Serous	Ov Ca	65.0	3	3	1	504	Complete response	12.1	12.1	54.0	Alive		Post	Carboplatinum	Taxol		Yes	2000	
29	1375	N (S)	0.004	Serous	Ov Ca	77.4	2	2	0	595	Complete response		54.2	54.2	Alive		Post	Carboplatinum			Yes	3000	
30	1385	Y	0.003	Serous	Ov Ca	77.6	3	3	miliary	NK	Partial response			17.6	Deceased	Unrelated to maligna	Post	Carboplatinum	Taxol		Yes		
31	1398	Y	ND	Serous	Ov Ca	64.6	3	3	miliary	3174	Complete response	13.5	13.5	42.3	Alive		Post	Carboplatinum	Taxol		Yes	2000	
32	1402	N	0.080	Serous	Ov Ca	49.4	2	3	0	1975	Complete response		48.8	48.8	Alive		Pre	Carboplatinum	Taxol		No		
33	1456	Y	0.004	Serous	Ov Ca	58.9	3	3	miliary	207	Complete response	8.3	8.3	16.6	Deceased	Related to malignanc	Post	Carboplatinum	Taxol		Yes	1000	
34	1463	Y	1.243	Serous	Ov Ca	54.0	3	2	miliary	320	Complete response	32.6	32.6	42.9	Alive		Peri	Carboplatinum	Taxol		No		
35	1466	N	0.024	Serous	Ov Ca	73.1	2	2	0	97	Complete response	29.5	29.5	43.0	Alive		Post	Carboplatinum			No		
36	1475	N	0.032	Serous	Ov Ca	75.9	3	2	miliary	153	Complete response	21.0	21.0	38.7	Alive		Post	Carboplatinum	Taxol		No		
37	1485	N	0.046	Serous	Ov Ca	53.7	3	3	miliary	206	Complete response	14.7	14.7	28.0	Deceased	Related to malignanc	Peri	Carboplatinum	Taxol		Yes	1500	
38	1487	Y	0.017	Serous	Ov Ca	58.0	3	3	<1	98	Complete response	18.2	18.2	43.0	Alive		Post	Carboplatinum			No		
39	1497	Y	0.024	Serous	Ov Ca	61.9	3	3	1	28300	Complete response	15.9	15.9	36.6	Alive		Post	Carboplatinum	Taxatere		No		
40	1521	Y	0.048	Serous	Ov Ca	59.9	3	3	0	199	Complete response	6.8	6.8	21.1	Alive		Post	Carboplatinum	Taxol		Yes		
41	1524	N (S)	0.233	Serous	Ov Ca	64.4	3	3	0	848	Partial response			26.0	Deceased	Related to malignanc	Post	Carboplatinum			No		
42	1527	N (S)	0.027	Serous	Ov Ca	60.0	4	3	>2	1496	Complete response	9.5	9.5	32.6	Alive		Post	Carboplatinum	Taxol		Yes	2000	
43	1528	N (S)	0.077	Serous	Ov Ca	56.3	3	2	miliary	2928	Complete response		29.6	29.6	Alive		Peri	Carboplatinum	Taxol		No		
44	1530	N (S)	0.004	Serous	Ov Ca	64.6	3	3	extensive	864	Partial response			18.0	Deceased	Related to malignanc	Post	Carboplatinum	Taxol		Yes		
45	1532	N (S)	0.032	Serous	Ov Ca	52.4	2	3	0	474	Complete response		28.4	28.4	Alive		Post	Carboplatinum	Taxol		No		
46	1535	Y	0.089	Serous	Ov Ca	44.9	3	3	>2	537	Complete response	9.9	9.9	29.6	Alive		Peri	Carboplatinum	Taxol		No		
47	1541	Y	0.027	Serous	Ov Ca	57.2	3	2	miliary	106	Complete response	17.1	17.1	29.3	Alive		Post	Carboplatinum	Taxol		No		
48	1543	Y	0.053	Serous	Ov Ca	72.8	3	2	<0.5	280	Partial response			25.8	Alive		Post	Carboplatinum			Yes		
49	1544	Y	0.010	Serous	Ov Ca	56.2	3	3	<2	1727	Complete response	11.5	11.5	33.8	Alive		Post	Carboplatinum			Yes	5000	
50	1552	N	0.073	Serous	Ov Ca	65.8	3	3	>2	228	Partial response			13.3	Alive		Post	Carboplatinum	Taxol		Yes		
51	1568	N (S)	0.020	Serous	Ov Ca	75.2	4	3	<2	2559	Partial response			22.4	Alive		Post	Carboplatinum			Unknown		
52	1635	Y	0.005	Serous	Ov Ca	62.0	1	2	0	66	Complete response	50.1	50.1	72.3	Alive		Post	Carboplatinum	Taxol		No		
53	1637	N	ND	Serous	Ov Ca	72.0	1	1	0	571	Complete response		20.8	20.8	Alive		Post	Nil			No		
54	1857	N	0.081	Serous	Ov Ca	76.4	3	3	>2	325	Complete response	14.9	14.9	43.0	Alive LFU		Post	Carboplatinum			No		
55	1904	N	0.025	Serous	Ov Ca	51.3	4	3	0	755	Complete response		8.2	8.2	Alive LFU		Peri	Carboplatinum	Taxol		No		
56	1914	N	0.004	Serous	Ov Ca	40.4	4	2	0.5	4399	Complete response	15.1	15.1	65.3	Alive		Pre	Carboplatinum	Taxol		Yes		
57	1917	N	0.008	Serous	Ov Ca	57.2	3	2	0.5	373	Complete response	17.0	17.0	39.0	Deceased	Related to malignanc	Post	Carboplatinum	Taxol		Yes	100	
58	1920	Y	0.258	Serous	Ov Ca	59.9	1	3	0	23170	Complete response		86.0	86.0	Alive		Post	Carboplatinum			No		
59	1931	N	0.125	Serous	Ov Ca	57.3	3	3	2	702	Partial response			54.0	Deceased	Related to malignanc	Post	Carboplatinum	Taxol		Yes		
60	1935	N	0.049	Serous	Ov Ca	63.4	3	3	<1	495	Complete response	15.7	15.7	27.5	Deceased	Related to malignanc	Post	Carboplatinum			Yes		
61	1977	N	0.002	Serous	Ov Ca	73.0	3	3	1.5	4440	Complete response	19.0	19.0	61.6	Alive		Post	Carboplatinum	Taxol		Yes	1000	
62	1983	N	0.124	Serous	Ov Ca	57.4	3	2	0.5	849	Complete response	16.7	16.7	56.2	Alive		Post	Carboplatinum	Taxol		Yes	2000	
63	1988	N	1.000	Serous	Ov Ca	49.6	1	1	0	47	Complete response		60.0	60.0	Alive LFU		Pre	Nil			Unknown		
64	2013	Y	0.012	Serous	Ov Ca	57.0	1	3	0	120	Complete response		20.6	20.6	Alive			Carboplatinum			No		
65	2021	Y (P)	0.046	Serous	Ov Ca	70.0	3	2	miliary	1200	Complete response		18.9	18.9	Alive		Post	Carboplatinum	Taxol		Unknown		
66	2045	Y	0.049	Serous	Ov Ca	59.0	3		0	7	Complete response	125.2	125.2	153.3	Alive		Post	Cisplatinum	Cyclophosphamide		Unknown		
67	2072	Y (P)	0.220	Serous	Ov Ca	24.0	3	2		NK	Complete response	16.1	16.1	42.0	Alive		Pre	Yes; unknown agents			Unknown		
68	3003	Y	0.051	Serous	Ov Ca	78.0	3	3	>2	2645	Partial response			5.5	Alive		Post	Carboplatinum			Yes	1500	
69	1000	N	0.088	Mucinous	Ov Ca	81.6	3	1	0.5	182	Complete response	19.9	19.9	21.0	Deceased	Related to malignanc	Post	Carboplatinum			Yes	5000	
70	1356	Y	0.418	Mucinous	Ov Ca	34.4	1	1	0	52	Complete response	11.0	11.0	16.0	Deceased	Related to malignanc	Pre	Nil			Yes	5000	
71	1567	Y	0.161	Mucinous	Ov Ca	51.7	2	1	0	1793	Complete response		25.8	25.8	Alive		Post	Carboplatinum	Taxol		No		
72	2016	Y	0.011	Mucinous	Ov Ca	44.4	3		0	95	Partial response			6.8	Alive		Pre	Carboplatinum	Taxol		No		
73	1155	Y	0.521	Endometriod	Ov Ca	66.6	1	1	0	NK	Complete response		41.9	41.9	Alive		Post	Nil			No		
74	1381	Y	0.273	Endometriod	Ov Ca	48.6	3	3	0	4669	Complete response	32.4	32.4	42.6	Alive		Post	Carboplatinum	Taxol	Gemcitabine	No		
75	1462	N	0.035	Endometriod	Ov Ca	49.4	3	3	miliary	503	Complete response	12.4	12.4	25.2	Alive LFU		Peri	Carboplatinum	Taxol		Yes	1000	
76	1547	N	0.842	Endometriod	Ov Ca	67.4	2	2	0	131	Complete response		24.1	24.1	Alive		Post	Carboplatinum			No		
77	2050	N	0.769	Endometriod	Ov Ca	52.8	1	1	0	76	Complete response		12.6	12.6	Alive		Post	Carboplatinum	Taxol		No		
78	1034	Y	0.025	Clear cell	Ov Ca	60.0	1		0	35	Complete response		43.8	43.8	Alive		Post	Nil			No		
79	1035	N	0.021	Clear cell	Ov Ca	38.3	3		0	152	Complete response	12.7	12.7	30.0	Deceased	Related to malignanc	Pre	Carboplatinum	Taxol	Topotecan	No		
80	1131	Y	0.018	Clear cell	Ov Ca	46.2	3		3	240	No response			3.0	Deceased	Related to malignanc	Pre	Carboplatinum	Taxol		Yes	2000	
81	1496	N	0.022	Clear cell	Ov Ca	32.6	2		0	715	Complete response	14.0	14	34.4	Alive		Pre	Carboplatinum	Taxol		No		
82	1804	Y	0.029	Clear cell	Ov Ca	57.5	3		1	611	Partial response			15.0	Deceased	Related to malignanc	Post	Carboplatinum	Taxol		No		
83		n=81	n=78																				
84																							
85	2005		13.150	NOSE																			
86	2002		0.778	NOSE																			
87	1644		0.843	NOSE																			
88	1641		0.929	NOSE																			
89	2009		0.544	NOSE																			
90	1642		0.693	NOSE																			
91	1640		0.469	NOSE																			
92	2008		0.739	NOSE																			
93	2019		0.923	NOSE																			
94	2034		0.466	NOSE																			
95	2042		3.590	NOSE																			
96	2090	N	2.930	NOSE																			
97	2082		7.677	NOSE																			
98	2089		12.872	NOSE																			

	A	B	C	D	E	F	G	H	I	J	K	L	M	N	O	P	Q	R	S	T	U	V	W
99	2023	Y		NOSE																			
100	2026	N		NOSE																			
101	2029	N		NOSE																			
102	2087	N		NOSE																			
103	1856	N		N Stroma																			
104	1910	N		N Stroma																			
105	1918	Y (P)		N Stroma																			
106	1933	N		N Stroma																			
107		n=9	n=14																				
108																							
109	Notes:																						
110	1. A description of residual disease as "miliary" (disseminated millet seed-like deposits) is treated as > 1cm, as determined by Kaplan Meier survival analysis																						
111	2. LFU = lost to further followup after the time indicated. These patients were censored in the outcome analysis.																						
112	3. Y = Ct<45 in 2/3 or 3/3 replicates																						
113	4. Y (P) = weak positive = 2/3 replicates giving signal but where 1 or 2 replicates are at Ct>45 OR if 3/3 replicates have Ct>45																						
114	5. N (S) = negative = positive signal by SYBER but not probe positive																						
115	6. N = No signal in all 3 replicates OR in only 1 replicate (<45 OR >45)																						
116	7. ND, not determined.																						
