# Supplementary figures and images for: Collagen and calcium-binding EGF domains 1 is frequently inactivated in ovarian cancer by aberrant promoter hypermethylation and modulates cell migration and survival
Source: Br J Cancer. 2009 Nov 24;102(1):87–96. doi: 10.1038/sj.bjc.6605429 (PMC2813742; doi:10.1038/sj.bjc.6605429)

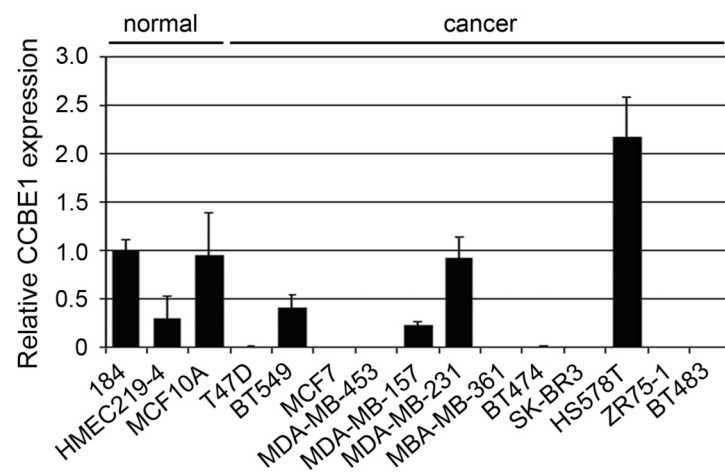

**a**

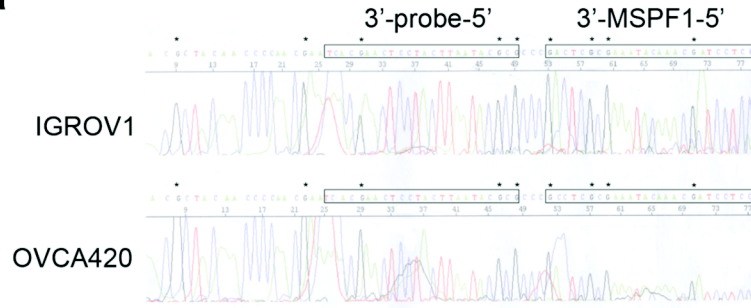**b**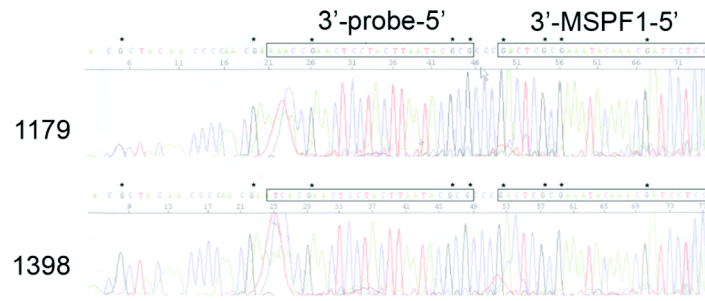

Supplement: Supplementary Figures 1–2 [file 6605429x2.pdf]
