# Supplementary material for: Collagen and calcium-binding EGF domains 1 is frequently inactivated in ovarian cancer by aberrant promoter hypermethylation and modulates cell migration and survival
Source: Br J Cancer. 2009 Nov 24;102(1):87–96. doi: 10.1038/sj.bjc.6605429 (PMC2813742; doi:10.1038/sj.bjc.6605429)
Supplement: Supplementary Figures 1–2 [file 6605429x3.doc]

. Methylation status of normal ovaries and primary ovarian carcinomas.

|  |  |
| --- | --- |
|  |  |
|  |  |
|  |  |
|  |  |
|  |  |
|  |  |
|  |  |

**Supplementary Data**

**Supplementary Table 1**. *CCBE1* expression and methylation results, and the corresponding clinical and pathological details of the patient cohort used in this study.

**Supplementary Figure 1**. Real-time quantitative PCR showing CCBE1 expression in a panel of normal breast and breast cancer cell lines. CCBE1 mRNA expression was normalised to GAPDH mRNA expression.

**Supplementary Figure 2**. **Direct sequencing of DNA to confirm methylation status.** A) Sequencing of bisulphite-treated genomic DNA isolated from IGROV1 and OVCA420 cell lines showing the methylation status of individual CpG sites in the promoter region of *CCBE1.* B) Sequencing of bisulphite-treated genomic DNA from two primary ovarian carcinomas confirming methylation at CpG sites in the promoter region of *CCBE1*. * indicates methylated CpG dinucleotides. Boxed regions indicate where the probe and MSP-F1 primer are located.
